# Current practices for respiratory syncytial virus surveillance across the EU/EEA Member States, 2017

**DOI:** 10.2807/1560-7917.ES.2019.24.40.1900157

**Published:** 2019-10-03

**Authors:** Madelief Mollers, Céline Barnadas, Eeva K Broberg, Pasi Penttinen, European Influenza Surveillance Network, Anne C Teirlinck, Thea K Fischer

**Affiliations:** 1 National Institute for Public Health and the Environment (RIVM) - Centre for Infectious disease control, Bilthoven, the Netherlands; 2 European Programme for Intervention Epidemiology Training (EPIET) and European Public Health Microbiology (EUPHEM) training programme, European Centre for Disease Prevention and Control (ECDC), Stockholm, Sweden; 3 These authors contributed equally to this manuscript; 4 Virus and Microbiological Special Diagnostics, Statens Serum Institut, Copenhagen, Denmark; 5 European Centre for Disease Prevention and Control, Stockholm, Sweden; 6 The members of the European Influenza Surveillance Network (EISN) are listed at the end of the article; 7 Department of Infectious Diseases and Centre for Global Health, University of Southern Denmark, Odense, Denmark; 8 These authors contributed equally to this manuscript

**Keywords:** RSV, respiratory syncytial virus, epidemiology, Europe, laboratory surveillance, respiratory infections, sentinel surveillance, non-sentinel surveillance, surveillance, survey

## Abstract

**Background:**

Respiratory syncytial virus (RSV) is a major contributor to lower respiratory tract infections worldwide and several vaccine candidates are currently in development. Following vaccine introduction, reliable RSV surveillance should enable monitoring of vaccination impact. Data on the RSV disease burden in the European Union and European Economic Area (EU/EEA) are sparse.

**Aim:**

The aim of this study was to gather knowledge on current practices of national RSV surveillance in the EU/EEA.

**Methods:**

National Coordinators and National Focal Points for Influenza (epidemiologists and virologists) from the EU/EEA countries (n = 31) were invited to participate in an online survey in August and September 2017. The questionnaire covered questions on epidemiological and laboratory aspects of RSV surveillance.

**Results:**

All EU/EEA countries except Liechtenstein replied to the survey. Eighteen countries reported to have a sentinel surveillance system, 26 countries a non-sentinel surveillance system and three countries to have neither. RSV data collection was mostly done within the context of influenza surveillance. A wide range of diagnostic and characterisation assays was used for the detection of RSV.

**Discussion:**

The majority of EU/EEA countries have some surveillance for RSV in place. The prevailing integration of RSV surveillance into the existing influenza sentinel surveillance system may lead to under-reporting of RSV. The documented variations in existing RSV surveillance systems and their outputs indicate that there is scope for developing guidelines on establishing comparable methods and outcomes for RSV surveillance across the EU/EEA, to ensure the availability of a consistent evidence base for assessing future vaccination programmes.

## Introduction

Respiratory syncytial virus (RSV) is a major contributor to lower respiratory tract infections (LRTI) worldwide [1]. It is estimated that RSV is responsible for 20 to 50 million cases of acute LRTI each year in children younger than 5 years, resulting in a large number of hospitalisations. Infants are affected more than other age groups, representing an estimated 45% of all hospital admissions and deaths from RSV [1]. The public health importance of RSV is due, at least in low-income settings, to its high morbidity and mortality in young children [2,3]. The disease burden among the elderly population is substantial as well, and may be similar to that of seasonal influenza A virus infection in some seasons [4-6].

Common symptoms of RSV infections in children usually include rhinorrhoea, cough, wheezing and low-grade fever. More severe presentations of RSV infections such as bronchiolitis, pneumonia and atypical extrapulmonary disease [7] can lead to hospitalisation. Comorbidities such as chronic lung and/or heart disease increase the risk of severe RSV disease. Importantly, natural immunity to RSV is not long-lasting which means that individuals are at risk of reinfections throughout their lifetime [8].

RSV is an enveloped virus, with a linear negative-sense single-stranded RNA genome, and belongs to the species *Human orthopneumovirus*. It contains 10 genes encoding 11 proteins, among which are the nucleoprotein (N), the glycoprotein (G) and the fusion protein (F). Laboratory detection of RSV infections uses antigen detection, nucleic acid amplification based assays and/or virus culture. RSV is divided into types A and B based on antigenic properties of the glycoprotein G. Both subtypes usually co-circulate during epidemic seasons, following an irregular, alternating prevalence pattern, subtype A having a higher cumulative prevalence than subtype B [9-12]. Most commonly, the G gene region is used for molecular typing. However, as the RSV genome is more variable than previously thought, genotyping by sequencing of selected genes or the whole genome is being investigated [13-15]. Repeated RSV infections are common and have been documented for both children and adults, while infections tend to be less severe after the first infection episode [16,17]. The host’s immune status and the antigenic properties of current circulating RSV strains compared with previous infecting strains are thought to be factors influencing the risk of reinfections [18].

To prevent RSV infection in high-risk groups such as infants with chronic lung or congenital heart disease and infants born preterm, it is recommended to administer a neutralising monoclonal antibody (Palivizumab) monthly in these groups as prophylaxis during the RSV season [19]. Monoclonal antibodies with extended half-life are in development [20]. Several RSV vaccine candidates are currently undergoing development and testing including live-attenuated vaccines and different types of constructs, e.g. particle-based and subunit vaccines as well as recombinant vectors. The first candidate in clinical trials to complete phase III evaluation was a pre-fusion F protein nanoparticle-based vaccine. The trial aimed to reduce the rate of medically significant RSV LRTI in infants in the first 90 days of life, but failed its primary endpoint (39% efficacy against medically significant RSV LRTI (97.5% confidence interval (CI): −1 to 64)), although an efficacy of 44% (95% CI: 20–62) against RSV LRTI hospitalisations was found [21]. Target populations for the different vaccines differ and include young children, older adults and pregnant women, and despite the failure of the above-mentioned vaccine, it is still expected that an RSV vaccine will be available in Europe in coming years [22,23]. 

To assess the impact of a future RSV vaccine, valid estimates of disease morbidity and mortality are essential. Also, generating data on RSV burden and identifying risk groups in the population is fundamental to raise awareness among the population, inform healthcare professionals and policymakers and support vaccine deployment. After introduction of RSV vaccination, a stable surveillance system will permit monitoring the effect of vaccination including vaccine effectiveness and impact on disease burden. Although data on RSV detections are available from several European countries [24], data on RSV disease burden within the European Union (EU) are at present still sparse [22]. There is currently no harmonised case definition or reporting system, which would be a prerequisite for comparison of surveillance data across regions and over time. In addition, uniform guidelines for RSV strain characterisation, including detailed sequence analysis and antigenic characterisation, are critical for the detection of changes in circulating strains following implementation of vaccination.

The objective of the present survey, conducted among all EU and European Economic Area (EEA) countries during August and September 2017, was to gather knowledge on the current practices of RSV epidemiological and laboratory surveillance. This knowledge could identify best practice and provide benchmarking data and thereby inform and strengthen RSV surveillance in the EU/EAA countries, particularly in view of the likely need for monitoring RSV vaccine effectiveness and impact in the future.

## Methods

The European Centre for Disease Prevention and Control (ECDC) National Coordinators, and National Focal and Operational Contact Points for Influenza from EU/EEA countries were invited to participate in the survey. The online questionnaire on the EUSurvey platform (https://ec.europa.eu/eusurvey/) covered questions on both epidemiological and laboratory aspects of RSV infection surveillance (Supplement 1). Epidemiologists and virologists from the national public health organizations responded to the epidemiology and laboratory-related sections of the survey, respectively.

### Online questionnaire 

The questionnaire was divided into five sections. The first section covered general questions about RSV surveillance in the country. The second and third sections covered questions related to sentinel and non-sentinel epidemiological RSV surveillance, respectively. Within each section, specific subsections allowed respondents to report detailed information on different systems (for example community surveillance by general practitioners (GPs) or hospital surveillance by paediatric intensive care units). The fourth section of the questionnaire related to laboratory identification of RSV and further characterisation of the virus into types A and B and genotypes, as well as laboratory capacity. Respondents were provided with a definition of the terms typing and genotyping used in the questionnaire: typing referred to the distinction of RSV types A and B and genotyping to characterisation of genotypes within A and B types. Respondents were asked about methods used for RSV detection and characterisation in diagnostic and surveillance laboratories. Respondents had the possibility to provide more than one answer, but an estimate of the number of laboratories performing each method was not requested. The fifth section addressed aspects of reporting and sharing RSV data. Each question comprised a comment box for further explanations. To allow more flexibility in filling the questionnaire, no question was mandatory and the epidemiological and laboratory parts of the questionnaire could be filled independently. 

### Study period 

The invitation to participate in the survey was sent out on 9 August 2017, with a final deadline for responding on 15 September 2017. Data from the online questionnaire was exported to, and analysed in Excel. Data analysis was performed in two phases. Following the initial data cleaning process, respondents were contacted again (October–November 2017) to clarify answers when necessary. 

### Ethical statement

Ethical approval was not needed for this survey.

## Results

### Response rate

All EU/EEA countries, except Liechtenstein (n = 30/31) responded to the questionnaire. In some countries, two institutions provided responses, for example Greece (National Influenza Centre (NIC)–Southern Greece and NIC–Northern Greece), the United Kingdom (UK) (England and Northern Ireland) and Spain (Valladolid National Influenza Center In Castile and Leon and Instituto de Salud Carlos III). 

### Sentinel surveillance

Sentinel surveillance systems are systems that have been set up for surveillance as primary goal. While they usually cover only a small part of the population, they are high-quality networks that systematically sample patients that fulfil the criteria in the case definition and are representative of the population. Of 30 countries, 18 reported to have a sentinel surveillance system that includes RSV: Austria, Belgium, Bulgaria, the Czech Republic, Denmark, Estonia, Finland, France, Germany, Hungary, Ireland, Latvia, the Netherlands, Poland, Portugal, Slovakia, Slovenia and the UK. Four of these countries reported the existence of multiple sentinel systems (Austria (n = 2), Belgium (n = 3), France (n = 2) and Slovakia (n = 2)). In total, 23 surveillance systems identified in 18 countries included RSV. Details on each system are provided in Supplement 2.

All sentinel surveillance systems, except for the RSV laboratory sentinel surveillance in Belgium and the hospital and GP surveillance in France, were part of influenza surveillance systems which were in most countries designed to capture patients with influenza-like-illness (ILI). All countries with sentinel surveillance systems had at least one system operated through GPs. Eleven countries reported GP practices as the operating health facility, seven other countries reported a combination of GPs with other practitioners such as school or family doctors, paediatricians or emergency unit. Four countries reported multiple sentinel systems. In addition to GP surveillance, Austria, France and Slovakia also had a system that was hospital-based and Belgium additionally reported hospital-based and laboratory-based systems.

Nine of 23 sentinel surveillance systems were active throughout the whole year and 12 systems during the influenza surveillance period (weeks 40–20). The reporting frequency was weekly for 21 systems. Reporters from 12 systems provided case-based data to the national level, nine provided aggregated data. For two systems, no information was filled out on the aggregation level of collected data. Five systems represented less than 1% of the population, four systems between 1% and 5% of the population, and four systems more than 5% of the population. Eligibility for sampling was specified for 16 systems: 10 used ILI cases, three combined ILI and acute respiratory infection (ARI), two used ARI only and two used severe acute respiratory infection (SARI).

### Non-sentinel surveillance

As opposed to sentinel surveillance, non-sentinel surveillance is mostly a passive surveillance system that collects notifications of reported cases or laboratory results. A denominator for the source population is not always provided. There were 26 countries with a non-sentinel surveillance system for RSV; Austria, Belgium, Bulgaria, Croatia, Cyprus, the Czech Republic, Denmark, Estonia, Finland, France, Germany, Greece, Hungary, Iceland, Ireland, Latvia, Malta, the Netherlands, Norway, Poland, Portugal, Romania, Spain, Slovenia, Sweden and the UK. Six of them reported two non-sentinel systems: Finland, Iceland, Ireland, the Netherlands, Poland and Sweden. In total, there were 32 non-sentinel surveillance systems in 26 countries; details on the individual systems are provided in Supplement 2). Of 32 non-sentinel surveillance systems, 16 were laboratory-based, 13 were hospital-based, one was both hospital-based and GP-based, and two were other systems.

Many countries reported that their non-sentinel surveillance system was part of influenza surveillance (13/32 reported systems), three were specifically for RSV, 14 were part of another surveillance system and two were RSV-related studies. Twenty-two surveillance systems that reported on the surveillance period were active throughout the whole year and eight during the influenza surveillance period (weeks 40–20). Of 23 systems for which data on reporting frequency was available, one reported monthly and 22 reported on a weekly basis. About half of the reported data were case-based (13/28 systems that reported on the aggregation level) compared with 15/28 systems that collected aggregated data. The percentage of the population covered by the non-sentinel system was reported as known in five of the systems. The number of tested specimens was known in 10 systems.

### Countries without respiratory syncytial virus surveillance

Three countries (Italy, Lithuania and Luxembourg) reported not having a surveillance system that includes RSV at the time of the survey (summer 2017). Of these countries, Italy and Luxembourg were interested in setting up a surveillance system that includes RSV in the next 3 years. Their preferred system would be GP- and laboratory-based surveillance. Luxembourg indeed introduced RSV surveillance for the northern hemisphere winter season 2018/19 by conducting RSV testing on samples collected from the influenza sentinel surveillance.

### Objectives of respiratory syncytial virus surveillance

The two most common objectives of RSV surveillance among the 22 countries responding to the questions about the objectives (Table 1) were to contribute to the overall understanding of the role of RSV in respiratory disease (n = 16/22) and to determine seasonality of RSV, monitor trends of RSV detections within and across RSV seasons and the impact of potential vaccination programmes per age and/or target group (n = 14/22). Additional objectives not presented in Table 1 were to collect information in order to prescribe prophylaxis (n = 1) and to measure overall incidence and trends (n = 1). Four countries did not report objectives of their surveillance system.

**Table 1:** Objectives and relevance of national respiratory syncytial virus surveillance, EU/EEA countries, September 2017

Chosen responses in the questionnaire	Number of responding countries
Objectives of national RSV surveillance (n = 22)	
Contribute to the overall understanding of the role of RSV in respiratory disease	16
Determine the seasonality of RSV, monitor trends of RSV detections within and across RSV seasons and the impact of potential vaccination programs per age/target group	14
Support the estimation of healthcare burden of RSV infection in the different age and target groups	9
Measure the impact of potential future RSV vaccination programmes (by collecting baseline data)	8
Track the prevalence of the two RSV types among circulating strains	5
Relevance of RSV surveillance (n = 30)	
Nationally	
Yes	25
No	3
Do not know	2
Internationally	
Yes	24
No	3
Do not know	3

EU/EEA: European Union/European Economic Area; RSV: respiratory syncytial virus.

The opinion of respondents of most countries was that RSV surveillance is relevant nationally (25 of 30 countries) as well as internationally (24 of 30 countries), see Table 1.

### Communication

Twenty-one countries provided information about communicating RSV surveillance data. Information about RSV surveillance is mostly shared with clinicians (19/21), public health professionals (18/21) and laboratories (17/21) but also with the public (15/21), the scientific community (10/21) and policymakers (10/21). One country did not communicate findings. Results were mostly communicated through national surveillance bulletins, websites and reports (19/20), scientific articles (11/20), media (6/20) and social media (4/20).

### Laboratory capacity

All 30 countries provided information on laboratory capacity at primary and national laboratory levels, and information by Member State reflects both sources (Table 2).

**Table 2:** Laboratory capacity for respiratory syncytial virus detection, typing and genotyping, EU/EEA, September 2017

Chosen responses in the questionnaire	Number of responding countries
Diagnostics of RSV at primary laboratory (n = 27)	
Real-time RT-PCR methods	22
Gel-based RT-PCR methods	2
Antigen detection (DFA, EIA, etc.)	13
Point of care test	9
Virus isolation	3
Other, including serology	3
Unknown	4
Primary diagnostics of RSV at the national laboratory (n = 28)	
Real-time RT-PCR methods	27
Gel-based RT-PCR methods	5
Antigen detection (DFA, EIA, etc.)	8
Rapid test	1
Virus isolation	9
Other, including serology	7
Unknown	0
Total number of RSV samples received and/or identified annually (n = 22)	
< 100	6
100–499	10
500–999	1
1,000–2,000	3
> 2,000	2
Target gene when performing typing (n = 20)	
N gene	13
G gene	2
F gene	4
Other (NxTAG RPP Luminex assay)	1
Unknown	5
Methods used for typing RSV (n = 15)	
One real-time RT-PCR for RSV A and B (with two probes for detection)	6
RSV A- and B-specific singleplex real-time RT-PCR	3
One RT-PCR for RSV A and B followed by sequencing	1
RSV A- and B-specific singleplex RT-PCR followed by sequencing	1
Other	5
Total number of RSV samples typed annually (n = 19)	
< 100	6
100–499	10
500–999	2
1,000–2,000	0
> 2,000	1
Success rate for RSV molecular typing (n = 18)	
≥ 80%	13
60–79%	1
40–59%	1
<40%	1
Unknown	2
Target gene when performing genotyping (n = 10)	
N gene	1
G gene	9
F gene	4
Unknown	2
Methods used for genotyping RSV (n = 9)	
RT-PCR and Sanger sequencing	9
Next generation sequencing	0
Other	0
Total number of RSV samples genotyped annually (n = 7)	
< 100	6
100–500	1
Success rate for RSV molecular genotyping (n = 9)	
>80%	4
60–80%	0
40–59%	0
<40%	0
Unknown	5
Planning to introduce new methods for RSV typing and/or genotyping in the future (n = 28)	
Next generation sequencing method	12
Sanger sequencing method	8
Real-time PCR assay	5
No	8
Unknown	3
Participation in quality assessment programmes for RSV detection or characterisation (n = 29)	
EQA (e.g. QCMD) for RSV detection and/or typing every year	19
EQA for RSV characterisation every year	2
Other (e.g. do not participate every year, etc.)	3
No	8
Unknown	0

DFA: direct immunofluorescence assay; EIA: enzyme immunoassay; EQA: external quality assessment; EU/EEA: European Union/European Economic Area; NxTAG RPP: NxTAG respiratory pathogen panel; QCMD: quality control for molecular diagnostics; RSV: respiratory syncytial virus.

#### Capacity at diagnostic laboratories

The respondents were asked about capacity for RSV detection in diagnostic laboratories across the country, based on their knowledge. Information on the number of laboratories performing such testing was provided by 22 countries (Table 2). In these countries, the number of laboratories performing RSV detection varied greatly from one to ca 60. Importantly, among all countries for which information was provided, all but one reported capacity for PCR-based detection of RSV at diagnostic laboratory level. In the country where PCR detection was not performed at diagnostic laboratories, the primary test used was antigen detection. Eleven countries reported availability of typing capacity for RSV A and B in some diagnostic laboratories.

#### Respiratory syncytial virus laboratory surveillance

Fifteen countries (Austria, Belgium, Bulgaria, the Czech Republic, Denmark, Finland, France, Germany, Ireland, Latvia, Portugal, Romania, Spain, Sweden and the UK) reported having a laboratory designated for RSV reference functions, while 13 reported not having such a laboratory. Eleven countries indicated that the reference laboratory received samples from sentinel and/or non-sentinel surveillance systems.

#### Technical capacity for respiratory syncytial virus detection, typing and genotyping at the central level

Twenty-seven of 28 countries performed a real-time RT-PCR based assay for RSV detection at the laboratory that responded to the survey (Table 2). In Hungary, assays based on antigen detection and virus isolation were used at the central laboratory to detect RSV (no information was provided regarding methods used at hospital diagnostic laboratories).

Seven countries reported not performing RSV typing. In the countries that performed RSV typing and provided information on the targeted gene (n = 15), the N gene was most often targeted (n = 13), with real-time RT-PCR being the predominant method. Among five countries using a method not listed in the questionnaire, four used a multiplex real-time RT-PCR that detected RSV-A and RSV-B together with other viruses, and one country, Ireland, used a PCR-based NxTAG Luminex multiplex assay.

Ten countries reported performing RSV genotyping. Nine countries provided information on the assays used for genotyping, which always included (but were not necessarily limited to) the G gene and a protocol involving Sanger sequencing (Table 2).

Sixteen of 22 laboratories in 19 countries received and/or identified fewer than 500 RSV-positive samples a year. Six countries provided information showing that all RSV-positive samples received at the central laboratory were typed (France, Latvia, the Netherlands, Portugal, Romania and Slovenia). Seven countries performed genotyping on a subset of detected RSV specimens (fewer than 100 annually for six of them). The reported success rate for typing was high, more than 80% in 13 of 16 responses. Four respondents only provided an estimate of their success rate for genotyping which indicated that when they performed genotyping, they had a high success rate. Nineteen countries intended to introduce typing and/or genotyping and to upgrade methods towards sequencing and next generation sequencing. Finally, two thirds of the respondents (17/29) indicated that their laboratory participated in external quality assessment (EQA) for the identification of RSV.

## Discussion

Of 30 responding EU/EEA countries, 27 reported having a surveillance system for RSV in place. Half of the countries had a sentinel surveillance system for RSV and 26 countries a non-sentinel surveillance system. These systems varied from very basic (e.g. aggregated data, limited clinical information) to more advanced (e.g. case-based, extensive clinical information). In all but one of the responding countries, RSV sentinel surveillance was part of influenza surveillance. Previous studies have shown that a RSV surveillance system could be built upon an existing influenza surveillance system [25,26]. Most sentinel surveillance systems in our survey were based on samples collected by GP practices while most non-sentinel systems were laboratory- or hospital-based.

The most common objective of the reported surveillance systems was to contribute to overall understanding of the role of RSV in respiratory disease. However, less than half of the countries reported disease burden or estimation of the impact of RSV vaccination programmes as an objective of their surveillance system. Understanding seasonality helps countries to make recommendations regarding the timing of the provision of monoclonal antibodies which are available for treatment and prophylaxis in the first year(s) of life, mainly for premature children [25,26]. If assessment of seasonality is the only or primary surveillance goal of a country, a non-sentinel surveillance system based on laboratory results or a subset of RSV cases may be sufficient. RSV-specific incidence data on less severe cases, i.e. patients that report to the GP or other primary care health facilities seems well-established in 60% of the European countries through primary care sentinel surveillance. Hospital surveillance will, however, be crucial to estimate the incidence of severe cases and to assess efficacy and effectiveness of RSV vaccines and monoclonal antibodies to prevent complications following infection. At the time of our survey, only four countries had an RSV sentinel hospital surveillance in place and would be able to provide such data, although a third of the countries indicated that measuring the impact of potential future vaccination programmes was a surveillance goal. Strengthening surveillance to detect more severe cases of RSV, e.g. as part of a SARI surveillance, should therefore be a main priority for countries aiming to assess vaccination impact. In order to produce reliable incidence data, denominator data will be essential in any of these systems.

A wide range of methods were used by diagnostic laboratories for the detection of RSV, with assays based on RT-PCR and antigen detection most frequently reported. However, no further information was collected on the use of RSV-specific vs multiplex respiratory virus diagnostic assays. As the use of RT-PCR over antigen detection for RSV in clinical settings has been increasing, the performance of surveillance systems has improved [27,28]. The variety of assays used may challenge comparison and generalisability of data across the EU/EAA and highlights the need for diagnostic laboratories to participate in an EQA for RSV detection.

While RSV surveillance was in place in 27 of the 30 surveyed countries, representing a clear increase from countries reporting to the European Influenza Surveillance System in 2007 [26], this study highlights the heterogeneity of these systems. For example, some countries had both a sentinel and non-sentinel system, had clear definitions for who should be sampled and/or collected additional clinical information, while others did not. National surveillance, based either on GP (community) visits or (paediatric) hospital and/or intensive care admissions, is crucial to monitor epidemic trends, define disease burden (e.g. to inform cost-effectiveness studies for available vaccines) and guide national decision making. Harmonisation of surveillance across the EU/EEA countries would have the additional value of enabling supranational analyses to improve the power of statistical analyses and to provide insights in geographic and demographic variations that cannot be observed on a national level [24]. Also, it should be discussed among the EU/EEA countries how RSV-surveillance should be ideally designed and how every country could contribute; it may not be necessary for every country to have the same system, but rather systems that complement each other on the European level.

In most countries, RSV surveillance was based on influenza sentinel surveillance systems and on samples from ILI or ARI cases. This has clear practical and financial advantages because the same sample can be used for both tests or logistics can be shared when different samples are used, and results of laboratory outcomes can be compared. However, it is important that adding an RSV component to influenza surveillance does not impair the current well-established influenza surveillance systems [29]. Currently, the WHO is performing a pilot study which aims to test whether it is feasible to make use of the Global Influenza Surveillance and Response System platform for RSV surveillance without adversely affecting the well-established ILI and SARI surveillance of influenza [30]. The first results of this pilot are encouraging and even indicated that combining RSV and influenza surveillance can be an advantage to the current influenza surveillance systems [31].

Often, both ILI case definitions at GP level and SARI definitions at hospital level include fever. These definitions are not sensitive enough for RSV cases, which can typically present with and without fever, and the sampling algorithms used in influenza sentinel sites may need to be adjusted. Therefore, the WHO RSV initiative has extended the ILI case definition to include also cases without fever [30]. A recent review of RSV cases reported through sentinel and non-sentinel sites in the EU/EEA showed clearly that most, if not all RSV cases are captured in the influenza surveillance period from week 40 to week 20 in the following year, while several influenza systems have year-round data [24]. However, that study also showed that RSV often starts circulating in or close to week 40, suggesting that very early onset of the RSV season can occur and would not be captured if surveillance is limited to the typical influenza surveillance period. In addition, not all sentinel systems may capture paediatric populations, if there is no specific focus on infants. Some countries systematically test samples from sentinel systems also for other respiratory viruses, notably by using published and/or adapted multiplex PCR assays [32,33]. These surveillance systems can be used efficiently to detect circulating respiratory viruses such as rhinovirus and also to rapidly set up surveillance for novel or re-emerging viruses such as enterovirus D68 [34,35].

The capacity to detect RSV using molecular methods was available in almost all EU/EEA countries. However, different assays were used and different genes targeted. Real-time PCR was reported to be the most commonly used assay and two thirds of the laboratories reported annual participation in an EQA, demonstrating their commitment to assuring quality of the results. Both for RSV A and RSV B, detection performance can vary considerably depending of the type of assay used; in particular real-time RT-PCR and nested RT-PCR performed better than conventional RT-PCR and commercial assays [36]. In a recent systematic review and meta-analysis, the sensitivity of rapid RSV antigen diagnostic tests was estimated at 74% and 88% when test results were compared with that of RT-PCR and immunofluorescence, respectively [37]. The survey conducted here was aimed mainly at providing information on laboratory capacity at central level where testing for active surveillance systems are performed; it did not permit to review in detail what capacity exists in passive surveillance systems using data from diagnostic laboratories in each country (e.g. percentage of diagnostic laboratories performing RSV detection or EQA).

Data on strains circulating before and after vaccine introduction will provide important information on the impact of vaccination as some vaccine candidates may impact the circulation of certain virus subtypes as is known for e.g. influenza vaccines and rotavirus vaccines. While genotyping can be at least partially achieved using Sanger sequencing, many countries reported to be willing and able to move towards next generation sequencing. Irrespective of the sequencing method, the time interval before deployment of effective vaccines provides an excellent opportunity to increase the general capacity for genotyping of RSV, including generation of new important knowledge about vaccine preventability at virus strain level. These efforts will also contribute to increasing general capacity for molecular epidemiology and surveillance at the European level.

## Conclusions

This survey demonstrates that almost all EU/EEA Member States have surveillance activities related to RSV. However, objectives of the surveillance vary between countries. In order to prepare for the possible introduction of RSV vaccines onto European markets, RSV surveillance should be strengthened and surveillance objectives need to be well-established. If the current primary aim is to determine seasonality, trends and to increase understanding of RSV, as pointed out as goals by the majority of responders, the surveillance systems should include both epidemiological and laboratory components and be applied as systematically as possible throughout the year. However, for the burden and risk group estimations needed before and after introducing vaccination programmes, more comprehensive studies would be required that are not necessarily continuous. The current pre-vaccination era should be used to obtain valid baselines for RSV burden and molecular epidemiological profiles, including the variability of antigenic sites targeted by vaccination, in order to effectively monitor post-immunisation changes. Once a vaccine is introduced, surveillance has an essential role to play in monitoring its impact and potential vaccine-induced changes in the circulating viruses and in informing policymakers.

## Members of the European Influenza Surveillance network (EISN)

Austria: Theresia Popow-Kraupp (Centre of Virology, Medical University Vienna)

Belgium: Nathalie Bossuyt, Isabelle Thomas and Cyril Barbezange (Sciensano)

Bulgaria: Neli Korsun (National Center of Infectious and Parasitic Diseases)

Croatia: Vladimir Drazenovic (Croatian National Institute of Public Health)

Cyprus: Maria Koliou and Despo Pieridou (Medical and Public Health Services, Ministry of Health)

Czech Republic: Jan Kyncl, Martina Havlickova and Ludmila Novakova (National Institute of Public Health)

Denmark: Ramona Trebbien (Statens Serum Institut)

Estonia: Olga Sadikova and Grethel Simonlatser (Health Board)

Finland: Outi Lyytikäinen, Niina Ikonen and Hanna Nohynek (Finnish Institute for Health and Welfare)

France: Emmanuel Belchior (Santé Publique France), Sylvie Behillil and Martine Valette (Centre National de Référence Virus des Infections Respiratoires Institut Pasteur Paris and Hospices Civils de Lyon)

Germany: Silke Buda, Brunhilde Schweiger, and Janine Reiche (Robert Koch Institute)

Greece: Anna Papa (Aristotle University of Thessaloniki) and Vasiliki Pogka and Andreas F. Mentis (Hellenic Pasteur Institute)

Hungary: Zsuzsanna Molnár and Mónika Rózsa (National Public Health Center (NPHC))

Iceland: Gudrún Sigmundsdóttir (Centre for Health Security and Communicable Disease Control, Directorate of Health in Iceland) and Gudrun Baldvinsdottir (Virology, Landspitali University Hospital, Reykjavik).

Ireland: Lisa Domegan and Joan O'Donnell (Health Protection Surveillance Centre - Health Service Executive); Allison Waters (National Virus Reference Laboratory, University College Dublin)

Italy: Caterina Rizzo (Unit of Innovation and Clinical Pathway, Bambino Gesù Children’s Hospital, Rome, Italy) and Maria Rita Castrucci (Istituto Superiore di Sanità)

Latvia: Raina Nikiforova (Centre for Disease Prevention and Control of Latvia), Natalija Zamjatina, Gatis Pakarna, Jelena Storozenko (RIGA east university hospital, Laboratory service, Latvian Centre of Infectious Diseases laboratory)

Lithuania: Asta Skrickiene (Centre for Communicable Diseases and AIDS) and Algirdas Griskevicius (National Public Health Surveillance Laboratory)

Luxembourg: Joël Mossong, Guillaume Fournier and Trung Nguyen (Laboratoire National de Santé)

Malta: Tanya Melillo (Infectious Disease Prevention and Control Unit) and Christopher Barbara (Pathology Department, Mater Dei Hospital)

The Netherlands: Adam Meijer (National Institute for Public Health and the Environment) and Gé A. Donker (Nivel, the Netherlands Institute for Health Services Research)

Norway: Siri Helene Hauge and Susanne G. Dudman (Norwegian Institute of Public Health)

Poland: Iwona Paradowska-Stankiewicz, Katarzyna Łuniewska and Lidia B. Brydak (National Institute of Public Health-National Institute of Hygiene)

Portugal: Ana Paula Rodrigues and Raquel Guiomar (National Institute of Health Dr Ricardo Jorge, Lisbon, Portugal)

Romania: Odette Popovici (National Institute of Public Health Romania- National Centre for Surveillance and Control of Communicable Diseases) and Alina Elena Ivanciuc (“Cantacuzino” National Institute of Research-Development for Microbiology and Immunology)

Slovakia: Adriana Mecochova and Edita Staronova (Public Health Authority of the Slovak Republic)

Slovenia: Maja Socan (National Institute of Public Health Slovenia) and Katarina Prosenc Trilar (National laboratory for health environment and food Slovenia)

Spain: Concepcion Delgado-Sanz, Jesús Oliva, (National Centre of Epidemiology, CIBERESP, Instituto de Salud Carlos III, Madrid); Francisco Pozo, Inmaculada Casas (National Centre of Microbiology, Instituto de Salud Carlos III, Madrid);

Sweden: Marie Rapp and Mia Brytting (The Public Health Agency of Sweden)

United Kingdom: Christopher Nugent (Public Health Agency Belfast), Muhammad Sartaj (Public Health Agency Northern Ireland), Richard Pebody and Joanna Ellis (Public Health England)

## Supplementary Data

198000 Supplement1

## Supplementary Data

430000 Supplement2

## References

[1] Shi T McAllister DA O’Brien KL Simoes EAF Madhi SA Gessner BD Global, regional, and national disease burden estimates of acute lower respiratory infections due to respiratory syncytial virus in young children in 2015: a systematic review and modelling study. Lancet. 2017;390(10098):946-58. 10.1016/S0140-6736(17)30938-8 28689664 PMC5592248

[2] Hall CB Weinberg GA Iwane MK Blumkin AK Edwards KM Staat MA The burden of respiratory syncytial virus infection in young children. N Engl J Med. 2009;360(6):588-98. 10.1056/NEJMoa0804877 19196675 PMC4829966

[3] Nair H Nokes DJ Gessner BD Dherani M Madhi SA Singleton RJ Global burden of acute lower respiratory infections due to respiratory syncytial virus in young children: a systematic review and meta-analysis. Lancet. 2010;375(9725):1545-55. 10.1016/S0140-6736(10)60206-1 20399493 PMC2864404

[4] Falsey AR Hennessey PA Formica MA Cox C Walsh EE Respiratory syncytial virus infection in elderly and high-risk adults. N Engl J Med. 2005;352(17):1749-59. 10.1056/NEJMoa043951 15858184

[5] Fleming DM Taylor RJ Lustig RL Schuck-Paim C Haguinet F Webb DJ Modelling estimates of the burden of Respiratory Syncytial virus infection in adults and the elderly in the United Kingdom. BMC Infect Dis. 2015;15(1):443. 10.1186/s12879-015-1218-z 26497750 PMC4618996

[6] Meijboom MJ Pouwels KB Luytjes W Postma MJ Hak E RSV vaccine in development: assessing the potential cost-effectiveness in the Dutch elderly population. Vaccine. 2013;31(52):6254-60. 10.1016/j.vaccine.2013.10.023 24148573

[7] Al-Maskari N Mohsin J Al-Maani A Al-Macki N Al-Ismaili S Atypical presentations of respiratory syncytial virus infection: case series. Sultan Qaboos Univ Med J. 2016;16(1):e86-91. 10.18295/squmj.2016.16.01.016 26909220 PMC4746050

[8] Russell CD Unger SA Walton M Schwarze J The human immune response to respiratory syncytial virus infection. Clin Microbiol Rev. 2017;30(2):481-502. 10.1128/CMR.00090-16 28179378 PMC5355638

[9] Peret TC Hall CB Schnabel KC Golub JA Anderson LJ Circulation patterns of genetically distinct group A and B strains of human respiratory syncytial virus in a community. J Gen Virol. 1998;79(Pt 9):2221-9. 10.1099/0022-1317-79-9-2221 9747732

[10] Anderson LJ Hierholzer JC Tsou C Hendry RM Fernie BF Stone Y Antigenic characterization of respiratory syncytial virus strains with monoclonal antibodies. J Infect Dis. 1985;151(4):626-33. 10.1093/infdis/151.4.626 2579169

[11] Yu J Liu C Xiao Y Xiang Z Zhou H Chen L Respiratory syncytial virus seasonality, Beijing, China, 2007-2015. Emerg Infect Dis. 2019;25(6):1127-35. 10.3201/eid2506.180532 31107230 PMC6537707

[12] Midulla F Nenna R Scagnolari C Petrarca L Frassanito A Viscido A How respiratory syncytial virus genotypes influence the clinical course in infants hospitalized for bronchiolitis. J Infect Dis. 2019;219(4):526-34. 10.1093/infdis/jiy496 30204889

[13] Pierangeli A Trotta D Scagnolari C Ferreri ML Nicolai A Midulla F Rapid spread of the novel respiratory syncytial virus A ON1 genotype, central Italy, 2011 to 2013. Euro Surveill. 2014;19(26):20843. 10.2807/1560-7917.ES2014.19.26.20843 25011065

[14] Reiche J Schweiger B Genetic variability of group A human respiratory syncytial virus strains circulating in Germany from 1998 to 2007. J Clin Microbiol. 2009;47(6):1800-10. 10.1128/JCM.02286-08 19386848 PMC2691087

[15] Martinelli M Frati ER Zappa A Ebranati E Bianchi S Pariani E Phylogeny and population dynamics of respiratory syncytial virus (Rsv) A and B. Virus Res. 2014;189:293-302. 10.1016/j.virusres.2014.06.006 24954788

[16] Ohuma EO Okiro EA Ochola R Sande CJ Cane PA Medley GF The natural history of respiratory syncytial virus in a birth cohort: the influence of age and previous infection on reinfection and disease. Am J Epidemiol. 2012;176(9):794-802. 10.1093/aje/kws257 23059788 PMC3481264

[17] Sande CJ Mutunga MN Okiro EA Medley GF Cane PA Nokes DJ Kinetics of the neutralizing antibody response to respiratory syncytial virus infections in a birth cohort. J Med Virol. 2013;85(11):2020-5. 10.1002/jmv.23696 23983183 PMC3798117

[18] Pangesti KNA Abd El Ghany M Walsh MG Kesson AM Hill-Cawthorne GA Molecular epidemiology of respiratory syncytial virus. Rev Med Virol. 2018;28(2):e1968. 10.1002/rmv.1968 29377415

[19] Groothuis JR Hoopes JM Hemming VG Prevention of serious respiratory syncytial virus-related illness. II: Immunoprophylaxis. Adv Ther. 2011;28(2):110-25. 10.1007/s12325-010-0101-y 21318605

[20] Zhu Q McLellan JS Kallewaard NL Ulbrandt ND Palaszynski S Zhang J A highly potent extended half-life antibody as a potential RSV vaccine surrogate for all infants. Sci Transl Med. 2017;9(388):eaaj1928. 10.1126/scitranslmed.aaj1928 28469033

[21] Novavax. Novavax announces topline results from phase 3 PrepareTM trial of ResVax for prevention of RSV disease in infants via maternal immunization. Rockville: Novavax. [Accessed:23 May 2019]. Available from: https://ir.novavax.com/news-releases/news-release-details/novavax-announces-topline-results-phase-3-preparetm-trial

[22] European Centre for Disease Prevention and Control (ECDC). Proceedings of the Workshop on burden of RSV disease in Europe: ECDC Expert Consultation Meeting Stockholm 23–24 November 2015. Stockholm: ECDC; [Accessed: 14 Jul 2018]. Available from: https://ecdc.europa.eu/sites/portal/files/media/en/press/events/Documents/Meeting%20report%20ECDC%20RSV%20surv%20and%20burden%20of%20disease%20workshop%2023-24%20Nov.pdf

[23] PATH. RSV vaccine and mAb snapshot. Vaccine resource library. Seattle: PATH; 2019. Available from: https://vaccineresources.org/details.php?i=1562

[24] Broberg EK Waris M Johansen K Snacken R Penttinen P European Influenza Surveillance Network Seasonality and geographical spread of respiratory syncytial virus epidemics in 15 European countries, 2010 to 2016. Euro Surveill. 2018;23(5):17-00284. 10.2807/1560-7917.ES.2018.23.5.17-00284 29409569 PMC5801642

[25] Meerhoff TJ Fleming D Smith A Mosnier A van Gageldonk-Lafeber AB Paget WJ Surveillance recommendations based on an exploratory analysis of respiratory syncytial virus reports derived from the European Influenza Surveillance System. BMC Infect Dis. 2006;6(1):128. 10.1186/1471-2334-6-128 16899110 PMC1560143

[26] Meerhoff TJ Mosnier A Schellevis F Paget WJ EISS RSV Task Group Progress in the surveillance of respiratory syncytial virus (RSV) in Europe: 2001-2008. Euro Surveill. 2009;14(40):19346. 19822120

[27] A Hogan C Caya C Papenburg J Rapid and simple molecular tests for the detection of respiratory syncytial virus: a review. Expert Rev Mol Diagn. 2018;18(7):617-29. 10.1080/14737159.2018.1487293 29890085

[28] Rabon-Stith KM McGuiness CB Saunders B Edelman L Kumar VR Boron ML Laboratory testing trends for respiratory syncytial virus, 2007-2011. J Clin Virol. 2013;58(3):575-8. 10.1016/j.jcv.2013.09.012 24103492

[29] World Health Organization (WHO). WHO technical meeting on piloting RSV Surveillance based on the Global Influenza Surveillance and Response System. 28-30 June 2016, Geneva, Switzerland. WHO/OHE/PED/GIP/2016.6. Geneva: WHO; 2016. Available from: https://www.who.int/influenza/resources/publications/Technical_Meeting_RSV_Pilot/en/

[30] World Health Organization (WHO). WHO global RSV surveillance pilot - objectives. Geneva: WHO; 2017. Available from: http://www.who.int/influenza/rsv/rsv_objectives/en/

[31] Broor S Campbell H Hirve S Hague S Jackson S Moen A Leveraging the Global Influenza Surveillance and Response System for global respiratory syncytial virus surveillance-opportunities and challenges. Influenza Other Respir Viruses. 2019;irv.12672.; Epub ahead of print. 10.1111/irv.12672 31444997 PMC7578328

[32] Coiras MT Pérez-Breña P García ML Casas I Simultaneous detection of influenza A, B, and C viruses, respiratory syncytial virus, and adenoviruses in clinical samples by multiplex reverse transcription nested-PCR assay. J Med Virol. 2003;69(1):132-44. 10.1002/jmv.10255 12436489

[33] Auburn H Zuckerman M Broughton S Greenough A Smith M Detection of nine respiratory RNA viruses using three multiplex RT-PCR assays incorporating a novel RNA internal control transcript. J Virol Methods. 2011;176(1-2):9-13. 10.1016/j.jviromet.2011.05.017 21620897

[34] Meijer A Benschop KS Donker GA van der Avoort HG Continued seasonal circulation of enterovirus D68 in the Netherlands, 2011-2014. Euro Surveill. 2014;19(42):20935. 10.2807/1560-7917.ES2014.19.42.20935 25358039

[35] Meijer A van der Sanden S Snijders BE Jaramillo-Gutierrez G Bont L van der Ent CK Emergence and epidemic occurrence of enterovirus 68 respiratory infections in The Netherlands in 2010. Virology. 2012;423(1):49-57. 10.1016/j.virol.2011.11.021 22177700

[36] Meerhoff TJ MacKay WG Meijer A Paget WJ Niesters HG Kimpen JL The impact of laboratory characteristics on molecular detection of respiratory syncytial virus in a European multicentre quality control study. Clin Microbiol Infect. 2008;14(12):1173-6. 10.1111/j.1469-0691.2008.02100.x 19046164

[37] Chartrand C Tremblay N Renaud C Papenburg J Diagnostic accuracy of rapid antigen detection tests for respiratory syncytial virus infection: systematic review and meta-analysis. J Clin Microbiol. 2015;53(12):3738-49. 10.1128/JCM.01816-15 26354816 PMC4652120

