# Supplementary material for: Current practices for respiratory syncytial virus surveillance across the EU/EEA Member States, 2017
Source: Euro Surveill. 2019 Oct 3;24(40):1900157. doi: 10.2807/1560-7917.ES.2019.24.40.1900157 (PMC6784450; doi:10.2807/1560-7917.ES.2019.24.40.1900157)
Supplement: Supplement1 [file 19-00157_TEIRLINCK_supplement1.pdf]

**Supplementary file 1:** Online questionnaire. This supplementary material is hosted by Eurosurveillance as supporting information alongside the article **Current practices for Respiratory Syncytial Virus surveillance across the EU/EEA Member States, 2017** on behalf of the authors who remain responsible for the accuracy and appropriateness of the content. The same standards for ethics, copyright, attributions and permissions as for the article apply. Eurosurveillance is not responsible for the maintenance of any links or email addresses provided therein.

# Respiratory syncytial virus surveillance, detection and typing survey to EU/EEA states

Fields marked with * are mandatory.

## INTRODUCTION AND RESPONDENT INFORMATION

Respiratory Syncytial Virus (RSV) is a known cause of substantial morbidity and mortality in children worldwide. Several new RSV vaccine candidates are currently undergoing development and testing. In the European region, RSV is not a notifiable disease which is why reliable data on RSV is generally sparse. Prior to implementation of vaccines, baseline data on RSV surveillance, detection and typing is required.

In the present survey, we aim to address the RSV surveillance, detection and typing practices around the European region, in order to provide an overview of the current RSV surveillance and identify gaps for the generation of reliable data. This survey is performed by fellows from the EPIET and EUPHEM programmes under the coordination of ECDC Influenza and other respiratory viruses programme.

In Europe, reporting systems, case-definitions and population-based surveillance are generally set by national policy. As RSV disease is not yet under EU-wide surveillance, there is no harmonised case definition or reporting system across countries. Therefore the main objectives of our questionnaire are as follows:

- What are the national objectives for RSV surveillance?
- Which data is collected on RSV in Europe?
- Which detection and typing methods are used?

This survey should be completed by the national RSV responsible epidemiologist and virologist or if not applicable, by the National Operational Contact Point for influenza (epidemiologist and virologist). The questionnaire can be filled in by several people, one at a time. During this process, the questionnaire can be saved as a draft. A web link for you to access your draft again will be created automatically. You can email it to yourself and/or your colleague epidemiologist, or virologist. It will take between 15 and 45 minutes to fill in the survey, depending on what is done for RSV surveillance in your country.

The survey contains the following sections:
- section 1 is on general background of RSV surveillance in your country
- section 2 and 3 are about the epidemiological parts of sentinel and non sentinel RSV surveillance
- section 4 is on the virological aspects of RSV surveillance
- section 5 has questions on the reporting possibilities for RSV surveillance

* **Which EU/EEA Member State are you responding on behalf of?**

- Austria
- Belgium
- Bulgaria
- Croatia
- Cyprus
- Czech Republic
- Denmark
- Estonia
- Finland
- France
- Germany
- Greece
- Hungary
- Iceland
- Ireland
- Italy
- Latvia
- Liechtenstein
- Lithuania
- Luxembourg
- Malta
- Netherlands
- Norway
- Poland
- Portugal
- Romania
- Slovakia
- Slovenia
- Spain
- Sweden
- United Kingdom

* **On behalf of which institute or region are you responding for?**

**Please provide your contact details in case we have further questions:**

**Epidemiologist:**

Name:

Function (e.g. leading respiratory epidemiologist, etc.):

Email:

Phone number:

**Virologist:**

Name:

Function (e.g. head of reference laboratory, etc.):

Email:

Phone number:

## SECTION 1: GENERAL QUESTIONS ON RSV SURVEILLANCE IN YOUR COUNTRY/REGION

**Is RSV notifiable in your country/region?**

- Yes
- No
- I do not know

Comments:

**Is there a RSV surveillance system in your country/region?**

- Yes
- No

Comments:

**Is there another RSV data collection system in your country/region?**

- ○ Yes
- ○ No

**What is the objective of RSV surveillance or other RSV data collection systems in your country?**

Multiple answers possible

- ☐ Measure the impact of potential future RSV vaccination programmes (by collecting baseline data)
- ☐ Determine the seasonality of RSV and monitor trends of RSV detections within and across RSV seasons and the impact of potential vaccination programmes per age/target group
- ☐ Track the prevalence of the two RSV types among circulating strains
- ☐ Support the estimation of healthcare burden of RSV infection in the different age and target groups
- ☐ Contribute to the overall understanding of the role of RSV in respiratory disease
- ☐ Other (please specify in the 'Comments' box below)

Comments:

**With whom does your organisation communicate RSV data?**

Multiple answers possible

- ☐ Policy makers
- ☐ Public health professionals
- ☐ Clinicians
- ☐ Laboratories
- ☐ Scientific community
- ☐ Public
- ☐ Other (please specify in the 'Comments' box below)
- ☐ We do not communicate the data
- ☐ I do not know

Comments:

**How do you communicate RSV data?**

Multiple answers possible

- ☐ Through national surveillance bulletins, websites or reports
- ☐ Through scientific articles
- ☐ Through media
- ☐ Through social media
- ☐ Other (please specify in the 'Comments' box below)
- ☐ I do not know

Comments:

**Would your country be interested to set up RSV surveillance in the next 3 years?**

- Yes
- No
- I do not know

Comments:

**If you are planning to introduce RSV surveillance in your country, which system will you be using?**

Multiple answers possible

- Primary care surveillance (please clarify in the 'Comments' box below)
- Hospital-based surveillance (please clarify in the 'Comments' box below)
- Laboratory surveillance (please clarify in the 'Comments' box below)
- Other (please clarify in the 'Comments' box below)

Comments:

**Are there national or clinical guidelines for who should be tested for RSV in primary care?**

- Yes – (please clarify and please share a link (below) or document (through email))
- No
- I do not know

Comments:

**Are there national or clinical guidelines for who should be tested for RSV in hospital care?**

- Yes – (please clarify and please share a link (below) or document (through email))
- No
- I do not know

Comments:

**Do you consider RSV surveillance relevant at national level?**

- Yes
- No
- I do not know

Comments:

**Do you consider RSV surveillance relevant at international level?**

- Yes
- No
- I do not know

Comments:

**Would your country be interested to participate in a European pilot on RSV surveillance?**

- Yes
- No
- I do not know

Comments:

## SECTION 2 : RSV SENTINEL SURVEILLANCE IN YOUR COUNTRY

**Is there a sentinel surveillance of RSV in your country**

Multiple answers possible

- [ ] Yes, through GPs (general practitioners)
- [ ] Yes, through hospitals
- [ ] Other (please specify in the 'Comments' box below)
- [ ] No

Comments:

**If there is a sentinel surveillance of RSV through hospitals in your country, could you please specify which wards are involved?**

Multiple answers possible

- [ ] Accident and emergency units
- [ ] Critical care units
- [ ] ICUs (intensive care units)
- [ ] PICUs (paediatric intensive care units)
- [ ] Paediatric departments
- [ ] Maternity departments
- [ ] Neonatal units
- [ ] Ear, nose and throat units
- [ ] Oncology wards
- [ ] Elderly services
- [ ] Other (please specify in the 'Comments' box below)

Comments:

You will now be asked a few questions regarding the non-sentinel surveillance in your country for each of the existing systems.

## Description of the sentinel surveillance of RSV through general practitioners

**Can you briefly describe the sentinel surveillance system through GPs in your country?**

Set up:

- ○ Specifically for RSV
- ○ Part of influenza surveillance
- ○ Part of other surveillance (please specify)

Period of surveillance:

- ○ Week 40 to week 20 (winter months)
- ○ All year round

Frequency of reporting

- ○ Weekly
- ○ Monthly

Aggregation level:

- ○ Case-based
- ○ Aggregated

Year of implementation

*between 1950 and 2017*

Please add a web link to a publication of the surveillance system if available (please write NA if not available)

Comments:

**What is the percentage of population that you target with the RSV sentinel surveillance through GPs?**

- 0-1%
- 1-5%
- 6-10%
- 11-20%
- 100%
- Other, if you know the specific target, please provide a number ___% in the 'Comments' box below
- I do not know

Comments:

**Is there a case definition used to identify RSV cases through the sentinel surveillance system through GPs?**

- Yes (please see below)
- No
- I do not know

Comments:

**Please provide your case definition of a confirmed RSV case, in this surveillance system, translated into English, in the text box. Please also provide a web link to your case definition if available:**

**Would your country be in a position to adjust case definitions in order to capture RSV cases more specifically?**

- ○ Yes (Please specify in the 'Comments' box below what would be most likely feasible)
- ○ No
- ○ I do not know

Comments:

**Do you have a sampling algorithm available for RSV in your country for the sentinel surveillance through GPs?**

- ○ Yes
- ○ No
- ○ I do not know

Comments:

**If you have a sampling algorithm available for RSV in your country for the sentinel surveillance through GPs, please describe the algorithm or provide a web link (or write NA if not available):**

**Which of the following data on RSV cases is available in the sentinel surveillance system through GPs in your country?**

Please tick all that apply

- ☐ Unique patient identifier
- ☐ Age or date of birth
- ☐ Sex
- ☐ Geographical information
- ☐ Date of clinical onset
- ☐ Date of sampling
- ☐ Date of diagnosis
- ☐ Date of notification to surveillance organisation
- ☐ Source of notification
- ☐ Clinical symptoms
- ☐ Immunosuppressive medication or condition
- ☐ Chronic lung disease
- ☐ Hospitalization
- ☐ Premature birth
- ☐ RSV related death
- ☐ Other pathogens detected
- ☐ Other, please specify in the 'Comments' box below
- ☐ I do not know

Comments:

## Description of the sentinel surveillance of RSV through hospital wards (excluding paediatric intensive care units (PICUs) - specific section dedicated)

**Can you briefly describe the sentinel surveillance system through hospital wards (except PICUs) in your country?**

Set up:
- ○ Specifically for RSV
- ○ Part of influenza surveillance
- ○ Part of other surveillance (please specify)

Period of surveillance:
- ○ Week 40 to week 20 (winter months)
- ○ All year round

Frequency of reporting
- ○ Weekly
- ○ Monthly

Aggregation level:
- ○ Case-based
- ○ Aggregated

Year of implementation

*between 1950 and 2017*

Please add a web link to a publication of the surveillance system if available (please write NA if not available)

Comments:

**What is the percentage of population that you target with the RSV sentinel surveillance through hospital wards (except PICUs)?**

- 0-1%
- 1-5%
- 6-10%
- 11-20%
- 100%
- Other, if you know the specific target, please provide a number ___% in the 'Comments' box below
- I do not know

Comments:

**Is there a case definition used to identify RSV cases through the sentinel surveillance system through hospital wards (non PICUs)?**

- Yes (please see below)
- No
- I do not know

Comments:

**Please provide your case definition of a confirmed RSV case, in this surveillance system, translated into English, in the text box. Please also provide a web link to your case definition if available:**

**Would your country be in a position to adjust case definitions in order to capture RSV cases more specifically?**

- Yes (Please specify in the 'Comments' box below what would be most likely feasible)
- No
- I do not know

Comments:

**Do you have a sampling algorithm available for RSV in your country for the sentinel surveillance through hospital wards (non PICUs)?**

- Yes
- No
- I do not know

Comments:

**If you have a sampling algorithm available for RSV in your country for the sentinel surveillance through hospital wards (non PICUs), please describe the algorithm or provide a web link (or write NA if not available):**

**Which of the following data on RSV cases is available in the sentinel surveillance system through hospital wards (non PICUs) in your country?**

Please tick all that apply

- ☐ Unique patient identifier
- ☐ Age or date of birth
- ☐ Sex
- ☐ Geographical information
- ☐ Date of clinical onset
- ☐ Date of sampling
- ☐ Date of diagnosis
- ☐ Date of notification to surveillance organisation
- ☐ Source of notification
- ☐ Clinical symptoms
- ☐ Immunosuppressive medication or condition
- ☐ Chronic lung disease
- ☐ Hospitalization
- ☐ Premature birth
- ☐ RSV related death
- ☐ Other pathogens detected
- ☐ Other, please specify in the 'Comments' box below
- ☐ I do not know

Comments:

## Description of the sentinel surveillance of RSV through paediatric intensive care units (PICUs) in the hospital

**Can you briefly describe the sentinel surveillance system through PICUs in your country?**

Set up:

- ○ Specifically for RSV
- ○ Part of influenza surveillance
- ○ Part of other surveillance (please specify)

Period of surveillance:

- Week 40 to week 20 (winter months)
- All year round

Frequency of reporting

- Weekly
- Monthly

Aggregation level:

- Case-based
- Aggregated

Year of implementation

*between 1950 and 2017*

Please add a web link to a publication of the surveillance system if available (please write NA if not available)

Comments:

**What is the percentage of population that you target with the RSV sentinel surveillance through PICUs?**

- 0-1%
- 1-5%
- 6-10%
- 11-20%
- 100%
- Other, if you know the specific target, please provide a number ___% in the 'Comments' box below
- I do not know

Comments:

**Is there a case definition used to identify RSV cases through the sentinel surveillance system through PICUs?**

- Yes (please see below)
- No
- I do not know

Comments:

**Please provide your case definition of a confirmed RSV case, in this surveillance system, translated into English, in the text box. Please also provide a web link to your case definition if available:**

**Would your country be in a position to adjust case definitions in order to capture RSV cases more specifically?**

- Yes (Please specify in the 'Comments' box below what would be most likely feasible)
- No
- I do not know

Comments:

**Do you have a sampling algorithm available for RSV in your country for the sentinel surveillance through PICUs?**

- Yes
- No
- I do not know

Comments:

**If you have a sampling algorithm available for RSV in your country for the sentinel surveillance through PICUs, please describe the algorithm or provide a web link (or write NA if not available):**

**Which of the following data on RSV cases is available in the sentinel surveillance system through PICUs in your country?**

Please tick all that apply

- ☐ Unique patient identifier
- ☐ Age or date of birth
- ☐ Sex
- ☐ Geographical information
- ☐ Date of clinical onset
- ☐ Date of sampling
- ☐ Date of diagnosis
- ☐ Date of notification to surveillance organisation
- ☐ Source of notification
- ☐ Clinical symptoms
- ☐ Immunosuppressive medication or condition
- ☐ Chronic lung disease
- ☐ Hospitalization
- ☐ Premature birth
- ☐ RSV related death
- ☐ Other pathogens detected
- ☐ Other, please specify in the 'Comments' box below
- ☐ I do not know

Comments:

## Description of the sentinel surveillance of RSV through another system?

**Can you briefly describe the sentinel surveillance system through another system in your country?**

Set up:

- ◯ Specifically for RSV
- ◯ Part of influenza surveillance
- ◯ Part of other surveillance (please specify)

Period of surveillance:

- ◯ Week 40 to week 20 (winter months)
- ◯ All year round

Frequency of reporting

- ◯ Weekly
- ◯ Monthly

Aggregation level:

- Case-based
- Aggregated

Year of implementation

*between 1950 and 2017*

Please add a web link to a publication of the surveillance system if available (please write NA if not available)

Comments:

**What is the percentage of population that you target with the RSV sentinel surveillance through another system?**

- 0-1%
- 1-5%
- 6-10%
- 11-20%
- 100%
- Other, if you know the specific target, please provide a number ___% in the 'Comments' box below
- I do not know

Comments:

**Is there a case definition used to identify RSV cases through the sentinel surveillance system through another system?**

- Yes (please see below)
- No
- I do not know

Comments:

**Please provide your case definition of a confirmed RSV case, in this surveillance system, translated into English, in the text box. Please also provide a web link to your case definition if available:**

**Would your country be in a position to adjust case definitions in order to capture RSV cases more specifically?**

- Yes (Please specify in the 'Comments' box below what would be most likely feasible)
- No
- I do not know

Comments:

**Do you have a sampling algorithm available for RSV in your country for the sentinel surveillance through another system?**

- Yes
- No
- I do not know

Comments:

**If you have a sampling algorithm available for RSV in your country for the sentinel surveillance through another system, please describe the algorithm or provide a web link (or write NA if not available):**

**Which of the following data on RSV cases is available in the sentinel surveillance system through another system in your country?**

Please tick all that apply

- ☐ Unique patient identifier
- ☐ Age or date of birth
- ☐ Sex
- ☐ Geographical information
- ☐ Date of clinical onset
- ☐ Date of sampling
- ☐ Date of diagnosis
- ☐ Date of notification to surveillance organisation
- ☐ Source of notification
- ☐ Clinical symptoms
- ☐ Immunosuppressive medication or condition
- ☐ Chronic lung disease
- ☐ Hospitalization
- ☐ Premature birth
- ☐ RSV related death
- ☐ Other pathogens detected
- ☐ Other, please specify in the 'Comments' box below
- ☐ I do not know

Comments:

## SECTION 3: NON-SENTINEL SURVEILLANCE

**Is there a non-sentinel surveillance of RSV in your country**

Multiple answers possible

- ☐ Yes, through GPs (general practitioners)
- ☐ Yes, through hospitals
- ☐ Yes, through laboratories
- ☐ Other (please specify in the 'Comments' box below)
- ☐ No

Comments:

**If there is a non-sentinel surveillance of RSV through hospitals in your country, could you please specify which wards are involved?**

Multiple answers possible

- ☐ Accident and emergency units
- ☐ Critical care units
- ☐ ICUs (intensive care units)
- ☐ PICUs (paediatric intensive care units)
- ☐ Paediatric departments
- ☐ Maternity departments
- ☐ Neonatal units
- ☐ Ear, nose and throat units
- ☐ Oncology wards
- ☐ Elderly services
- ☐ Other (please specify in the 'Comments' box below)

Comments:

**You will now be asked a few questions regarding the non-sentinel surveillance in your country for each of the existing systems.**

## *Description of the non-sentinel surveillance of RSV through general practitioners*

**Can you briefly describe the non-sentinel surveillance system through GPs in your country?**

Set up:

- ◯ Specifically for RSV
- ◯ Part of influenza surveillance
- ◯ Part of other surveillance (please specify)

Period of surveillance:

- ◯ Week 40 to week 20 (winter months)
- ◯ All year round

Frequency of reporting

- ◯ Weekly
- ◯ Monthly

Aggregation level:

- ◯ Case-based
- ◯ Aggregated

Year of implementation

*between 1950 and 2017*

Please add a web link to a publication of the surveillance system if available (please write NA if not available)

Comments:

**Is there a case definition used to identify RSV cases through the non-sentinel surveillance system through GPs?**

- Yes
- No
- I do not know

Comments:

**Please provide your case definition of a confirmed RSV case, translated into English, in the text box. Please also provide a web link to your case definition if available:**

**Would your country be in a position to adjust case definitions in order to capture RSV cases more specifically?**

- Yes (Please specify in the 'Comments' box below what would be most likely feasible)
- No
- I do not know

Comments:

**Which of the following data on RSV cases is available in the non-sentinel surveillance system through GPs in your country?**

Please tick all that apply

- [ ] Unique patient identifier
- [ ] Age or date of birth
- [ ] Sex
- [ ] Geographical information
- [ ] Date of clinical onset
- [ ] Date of sampling
- [ ] Date of diagnosis
- [ ] Date of notification to surveillance organisation
- [ ] Source of notification
- [ ] Clinical symptoms
- [ ] Immunosuppressive medication or condition
- [ ] Chronic lung disease
- [ ] Hospitalization
- [ ] Premature birth
- [ ] RSV related death
- [ ] Other pathogens detected
- [ ] Other, please specify in the 'Comments' box below
- [ ] I do not know

Comments:

**Is the total number of RSV tests performed in the context of the non-sentinel surveillance through GPs (denominator) known?**

- ○ Yes
- ○ No
- ○ I do not know

Comments:

## *Description of the non-sentinel surveillance of RSV through hospital wards (excluding paediatric intensive care units (PICUs) - specific section dedicated)*

**Can you briefly describe the non-sentinel surveillance system through hospital wards (non PICUs) in your country?**

Set up:

- Specifically for RSV
- Part of influenza surveillance
- Part of other surveillance (please specify)

Period of surveillance:

- Week 40 to week 20 (winter months)
- All year round

Frequency of reporting

- Weekly
- Monthly

Aggregation level:

- Case-based
- Aggregated

Year of implementation

*between 1950 and 2017*

Please add a web link to a publication of the surveillance system if available (please write NA if not available)

Comments:

**Is there a case definition used to identify RSV cases through the non-sentinel surveillance system through hospital wards (non PICUs)?**

- Yes
- No
- I do not know

Comments:

**Please provide your case definition of a confirmed RSV case, translated into English, in the text box. Please also provide a web link to your case definition if available:**

**Would your country be in a position to adjust case definitions in order to capture RSV cases more specifically?**

- Yes (Please specify in the 'Comments' box below what would be most likely feasible)
- No
- I do not know

Comments:

**Which of the following data on RSV cases is available in the non-sentinel surveillance system through hospital wards (non PICUs) in your country?**

Please tick all that apply

- Unique patient identifier
- Age or date of birth
- Sex
- Geographical information
- Date of clinical onset
- Date of sampling
- Date of diagnosis
- Date of notification to surveillance organisation
- Source of notification
- Clinical symptoms
- Immunosuppressive medication or condition
- Chronic lung disease
- Hospitalization
- Premature birth
- RSV related death
- Other pathogens detected
- Other, please specify in the 'Comments' box below
- I do not know

Comments:

**Is the total number of RSV tests performed in the context of the non-sentinel surveillance through hospital wards (non PICUs) (denominator) known?**

- Yes
- No
- I do not know

Comments:

## *Description of the non-sentinel surveillance of RSV through paediatric intensive care units (PICUs)*

**Can you briefly describe the non-sentinel surveillance system through PICUs in your country?**

Set up:
- Specifically for RSV
- Part of influenza surveillance
- Part of other surveillance (please specify)

Period of surveillance:
- Week 40 to week 20 (winter months)
- All year round

Frequency of reporting
- Weekly
- Monthly

Aggregation level:
- Case-based
- Aggregated

Year of implementation

*between 1950 and 2017*

Please add a web link to a publication of the surveillance system if available (please write NA if not available)

Comments:

**Is there a case definition used to identify RSV cases through the non-sentinel surveillance system through PICUs?**
- Yes
- No
- I do not know

Comments:

**Please provide your case definition of a confirmed RSV case, translated into English, in the text box. Please also provide a web link to your case definition if available:**

**Would your country be in a position to adjust case definitions in order to capture RSV cases more specifically?**

- ○ Yes (Please specify in the 'Comments' box below what would be most likely feasible)
- ○ No
- ○ I do not know

Comments:

**Which of the following data on RSV cases is available in the non-sentinel surveillance system through PICUs in your country?**

Please tick all that apply

- ☐ Unique patient identifier
- ☐ Age or date of birth
- ☐ Sex
- ☐ Geographical information
- ☐ Date of clinical onset
- ☐ Date of sampling
- ☐ Date of diagnosis
- ☐ Date of notification to surveillance organisation
- ☐ Source of notification
- ☐ Clinical symptoms
- ☐ Immunosuppressive medication or condition
- ☐ Chronic lung disease
- ☐ Hospitalization
- ☐ Premature birth
- ☐ RSV related death
- ☐ Other pathogens detected
- ☐ Other, please specify in the 'Comments' box below
- ☐ I do not know

Comments:

**Is the total number of RSV tests performed in the context of the non-sentinel surveillance through PICUs (denominator) known?**

- ○ Yes
- ○ No
- ○ I do not know

Comments:

## *Description of the non-sentinel surveillance of RSV through laboratories*

**Can you briefly describe the non-sentinel surveillance system through laboratories in your country?**

Set up:

- Specifically for RSV
- Part of influenza surveillance
- Part of other surveillance (please specify)

Period of surveillance:

- Week 40 to week 20 (winter months)
- All year round

Frequency of reporting

- Weekly
- Monthly

Aggregation level:

- Case-based
- Aggregated

Year of implementation

*between 1950 and 2017*

Please add a web link to a publication of the surveillance system if available (please write NA if not available)

Comments:

**Is a case definition used to identify RSV cases through the non-sentinel surveillance system through laboratories?**

- Yes (please see below)
- No
- I do not know

Comments:

**Please provide your case definition of a confirmed RSV case, translated into English, in the text box. Please also provide a web link to your case definition if available:**

**Would your country be in a position to adjust case definitions in order to capture RSV cases more specifically?**

- ○ Yes (Please specify in the 'Comments' box below what would be most likely feasible)
- ○ No
- ○ I do not know

Comments:

**Which of the following data on RSV cases is available in the non-sentinel surveillance system through laboratories in your country?**

Please tick all that apply

- ☐ Unique patient identifier
- ☐ Age or date of birth
- ☐ Sex
- ☐ Geographical information
- ☐ Date of clinical onset
- ☐ Date of sampling
- ☐ Date of diagnosis
- ☐ Date of notification to surveillance organisation
- ☐ Source of notification
- ☐ Clinical symptoms
- ☐ Immunosuppressive medication or condition
- ☐ Chronic lung disease
- ☐ Hospitalization
- ☐ Premature birth
- ☐ RSV related death
- ☐ Other pathogens detected
- ☐ Other, please specify in the 'Comments' box below
- ☐ I do not know

Comments:

**Is the total number of RSV tests performed in the context of the non-sentinel surveillance (denominator) known?**

- Yes
- No
- I do not know

Comments:

## *Description of the non-sentinel surveillance of RSV through another system*

**Can you briefly describe the 'other' non-sentinel surveillance system in your country?**

Set up:

- Specifically for RSV
- Part of influenza surveillance
- Part of other surveillance (please specify)

Period of surveillance:

- Week 40 to week 20 (winter months)
- All year round

Frequency of reporting

- Weekly
- Monthly

Aggregation level:

- Case-based
- Aggregated

Year of implementation

*between 1950 and 2017*

Please add a web link to a publication of the surveillance system if available (please write NA if not available)

Comments:

**Is a case definition used to identify RSV cases through the non-sentinel surveillance system?**

- Yes (please see below)
- No
- I do not know

Comments:

**Please provide your case definition of a confirmed RSV case, translated into English, in the text box. Please also provide a web link to your case definition if available:**

**Would your country be in a position to adjust case definitions in order to capture RSV cases more specifically?**

- Yes (Please specify in the 'Comments' box below what would be most likely feasible)
- No
- I do not know

Comments:

**Which of the following data on RSV cases is available in the 'other' non-sentinel surveillance system in your country?**

Please tick all that apply

- [ ] Unique patient identifier
- [ ] Age or date of birth
- [ ] Sex
- [ ] Geographical information
- [ ] Date of clinical onset
- [ ] Date of sampling
- [ ] Date of diagnosis
- [ ] Date of notification to surveillance organisation
- [ ] Source of notification
- [ ] Clinical symptoms
- [ ] Immunosuppressive medication or condition
- [ ] Chronic lung disease
- [ ] Hospitalization
- [ ] Premature birth
- [ ] RSV related death
- [ ] Other pathogens detected
- [ ] Other, please specify in the 'Comments' box below
- [ ] I do not know

Comments:

**Is the total number of RSV tests performed in the context of the 'other' non-sentinel surveillance (denominator) known?**

- ○ Yes
- ○ No
- ○ I do not know

Comments:

# SECTION 4: VIROLOGICAL ASPECTS (RSV DETECTION AND TYPING) IN SENTINEL AND NON-SENTINEL SURVEILLANCE

## *Questions relating to the primary diagnostics of RSV*

**How many laboratories perform RSV detection in your country?**

Please fill number or "I don't know"

**Which method(s) are commonly used for RSV detection?**

| | Antigen detection (DFA, EIA, etc.) | Point of care test | Gel-based RT-PCR methods | Real-time RT-PCR methods | Virus isolation (specify cell lines used) | Other (including serology, please specify) | I do not know |
|---|---|---|---|---|---|---|---|
| At diagnostic laboratories | ☐ | ☐ | ☐ | ☐ | ☐ | ☐ | ☐ |
| At the national laboratory | ☐ | ☐ | ☐ | ☐ | ☐ | ☐ | ☐ |

Comments- Please also specify here if you are using commercial and/or in house assays:

## *Questions relating to the characterisation/typing of RSV viruses*

In this section, we refer to typing for the characterisation of RSV subtypes A and B, and genotyping for further characterisation of genotypes that belong to those subtypes.

**Do you have a laboratory designated for RSV reference functions, e.g. typing and characterisation in your country?**

- Yes
- No
- I do not know

Comments:

**If there is a laboratory designated for RSV reference functions, and if it is not your institute, please provide details of that laboratory:**

**Is typing (and/or genotyping) performed at diagnostic laboratories in your country?**

- Yes
- No
- I do not know

Comments:

**Of the diagnostic laboratories, how many send samples to the national (or regional) level:**

**for typing?**

Please fill number or "I don’t know"

**for further characterisation (genotyping)?**

Please fill number or "I don’t know"

Comments:

**Which RSV-positive specimens are submitted and selected for further typing and genotyping?**

| | Specimens submitted through the sentinel surveillance system | Specimens submitted through the non-sentinel surveillance system | Other | I do not know |
|---|---|---|---|---|
| Typing all samples | ☐ | ☐ | ☐ | ☐ |
| Typing selected samples | ☐ | ☐ | ☐ | ☐ |
| Genotyping all samples | ☐ | ☐ | ☐ | ☐ |
| Genotyping selected samples | ☐ | ☐ | ☐ | ☐ |

**If you have ticked 'Other' at the previous question, could you please specifiy which samples are typed and/or genotyped?**

Comments:

**Which target gene is used at your laboratory for typing and genotyping of RSV?**

| | G gene (glycoprotein) | F gene (fusion protein) | N gene (nucleocapsid) | Other (please specify in the 'Comments' box below) | I do not know | We do not perform |
|---|---|---|---|---|---|---|
| Typing | ☐ | ☐ | ☐ | ☐ | ☐ | ☐ |
| Genotyping | ☐ | ☐ | ☐ | ☐ | ☐ | ☐ |

Comments:

* **Which of the following methods is used at your laboratory for typing of RSV?**

- One broad real-time RT-PCR for RSV-A and -B (with two probes for detection)
- RSV-A and B specific singleplex real-time RT-PCR
- One broad RT-PCR for RSV-A and -B followed by sequencing
- RSV-A and RSV-B specific singleplex RT-PCR followed by sequencing
- Other (please specify, see below)
- I do not know

Comments:

**Please give the references of the assay used for typing or provide primer sequences:**

* **Which method is used at your laboratory for genotyping of RSV?**

- RT-PCR and Sanger sequencing
- Next generation sequencing
- Other (please specify, see below)
- I do not know

Comments:

**Please give the references of the assay used for genotyping or provide primer sequences:**

## *Questions related to the capacity of your laboratory*

**Please estimate the number of RSV samples identified subjected for typing and genotyping annually in your laboratory:**

| | Less than 100 | 100-500 | 500-1000 | 1000-2000 | More than 2000 |
|---|---|---|---|---|---|
| Total no. RSV samples received and /or identified | ☐ | ☐ | ☐ | ☐ | ☐ |
| Total no. RSV samples typed | ☐ | ☐ | ☐ | ☐ | ☐ |
| Total no. RSV samples genotyped | ☐ | ☐ | ☐ | ☐ | ☐ |

**If you know the exact numbers for 2016, please state:**

Total no. RSV samples received:

*between 1 and 50000*

No. RSV samples typed:

*between 1 and 50000*

No. RSV samples genotyped:

*between 1 and 50000*

Comments:

**Please estimate your success rate for RSV molecular typing and genotyping:**

| | Over 80% of processed RSV samples | 60-80% of processed RSV samples | 40-60% of processed RSV samples | Less than 40% of processed RSV samples | I do not know |
|---|---|---|---|---|---|
| Typing | ☐ | ☐ | ☐ | ☐ | ☐ |
| Genotyping | ☐ | ☐ | ☐ | ☐ | ☐ |

Comments:

**Are you planning to introduce new methods for RSV subtyping and/or genotyping in the future?**

- [ ] Yes – real-time PCR assay
- [ ] Yes – Sanger Sequencing method
- [ ] Yes – Next Generation Sequencing method
- [ ] Yes – Something else
- [ ] No
- [ ] I do not know

**If you are you planning to introduce new methods for RSV subtyping and/or genotyping in the future, please state date and specifiy which assays:**

Comments:

**Do you participate in quality assessment program/s for RSV detection or characterisation?**

- [ ] Yes, we participate in an EQA (e.g. QCMD) for RSV detection every year (please specify the EQA programme)
- [ ] Yes, we participate in an EQA for RSV characterisation every year (please specify the EQA programme)
- [ ] Other (e.g. don't participate every year, etc. - please specify in the 'Comments' box below)
- [ ] No
- [ ] I do not know

Comments:

## SECTION 5: REPORTING DATA

**Are the sentinel data as described above available for reporting to international level and are you willing to share these data?**

- ( ) Yes
- ( ) Only a subset, please specify in the 'Comments' box below
- ( ) No

Comments:

**What is the level of aggregation of the sentinel data that you would be able to share:**

| | Case based data with demographic, clinical and virological details | Aggregated by detections | Aggregated by types | Aggregated by age group | Not applicable |
|---|---|---|---|---|---|
| Surveillance through GPs | ☐ | ☐ | ☐ | ☐ | ☐ |
| Surveillance through paediatric intensive care units (PICUs) | ☐ | ☐ | ☐ | ☐ | ☐ |
| Surveillance through other hospital wards | ☐ | ☐ | ☐ | ☐ | ☐ |
| Surveillance through another system | ☐ | ☐ | ☐ | ☐ | ☐ |

Comments:

**Are the non-sentinel data as described above available for reporting to international level and are you willing to share these data?**

- ○ Yes
- ○ Only a subset, please specify in the 'Comments' box below
- ○ No

Comments:

**What is the level of aggregation of the non-sentinel data that you would be able to share:**

| | Case based data with demographic, clinical and virological details | Aggregated by detections | Aggregated by types | Aggregated by age group | Not applicable |
|---|---|---|---|---|---|
| Surveillance through GPs | ☐ | ☐ | ☐ | ☐ | ☐ |
| Surveillance through paediatric intensive care units (PICUs) | ☐ | ☐ | ☐ | ☐ | ☐ |
| Surveillance through other hospital wards | ☐ | ☐ | ☐ | ☐ | ☐ |
| Surveillance through another system | ☐ | ☐ | ☐ | ☐ | ☐ |

Comments:

**What data on RSV could you provide for years 2014, 2015 and 2016?**

| | 2014 | 2015 | 2016 |
|---|---|---|---|
| The total number of RSV positive samples detecte | ☐ | ☐ | ☐ |
| Information on the population under surveillance | ☐ | ☐ | ☐ |
| The detections by RSV type only | ☐ | ☐ | ☐ |
| The detections by RSV type and number of specimen tested (denominators) | ☐ | ☐ | ☐ |
| The detections by RSV type and number of specimen tested (denominators) in different age categories | ☐ | ☐ | ☐ |
| Information of RSV genotypes (sequences) | ☐ | ☐ | ☐ |
| Other (please state) | ☐ | ☐ | ☐ |

Comments:

## END OF THE SURVEY

**Thank you for your participation.**

**If you have any questions relating to this survey, please do not hesitate to contact** xxxxx

**The summary of this questionnaire will be circulated by the end of September 2017.**

**If you would like to make further comments, please use the box below:**
