# Supplementary material for: Current practices for respiratory syncytial virus surveillance across the EU/EEA Member States, 2017
Source: Euro Surveill. 2019 Oct 3;24(40):1900157. doi: 10.2807/1560-7917.ES.2019.24.40.1900157 (PMC6784450; doi:10.2807/1560-7917.ES.2019.24.40.1900157)
Supplement: Supplement2 [file 19-00157_TEIRLINCK_supplement2.pdf]

Supplementary file 2: Country specific RSV surveillance in EU/EEA Member States

This supplementary material is hosted by *Eurosurveillance* as supporting information alongside the article **Current practices for Respiratory Syncytial Virus surveillance across the EU/EEA Member States, 2017**, on behalf of the authors, who remain responsible for the accuracy and appropriateness of the content. The same standards for ethics, copyright, attributions and permissions as for the article apply. Supplements are not edited by Eurosurveillance and the journal is not responsible for the maintenance of any links or email addresses provided therein.

Austria

	**Sentinel**		**Non-sentinel**
**Type of health facilities**	GPs and Paediatricians	Hospitals (PICU and paediatric departments)	Labs (notifications)
**Set up**	Part of influenza surveillance	Part of influenza surveillance	Part of influenza surveillance
**Period**	Week 40-20	Week 40-20	Week 40-20
**Frequency**	Weekly	Weekly	Weekly
**Aggregation level**	Case-based	Case-based	Aggregated
**Population covered**	0-1%	0-1%	x
**Year introduced**	2000	2000	2012
**Eligible for sampling**	ILI/ARI	SARI	No
**Sampling algorithm for RSV**	No	No	No
**Information available**	Unique patient identifier;Age or date of birth;Sex;Geographical information;Date of clinical onset;Date of sampling;Date of diagnosis;Clinical symptoms;Immunosuppressive medication or condition;Chronic lung disease;Other pathogens detected	Unique patient identifier;Age or date of birth;Sex;Geographical information;Date of clinical onset;Date of sampling;Date of diagnosis;Clinical symptoms;Immunosuppressive medication or condition;Chronic lung disease;Hospitalization	Reporting of aggregated data
**Denominator known**			No

Belgium

	Sentinel			Non-sentinel
**Type of health facilities**	GPs	Hospital	Labs	Hospitals sent samples to national reference lab for typing
**Set up**	Part of influenza surveillance	Part of influenza surveillance	Part of other surveillance	Part of other surveillance
**Period**	Week 40 to 20	Week 40 to 20	All year round	All year round
**Frequency**	Weekly	Weekly	Weekly	
**Aggregation level**	Case-based	Case-based	Case-based	Case-based
**Population covered**			~60 of all microbiological labs	
**Year introduced**	Season 2015-2016	Season 2015-2016	1996	2013
**Eligible for sampling**	ILI	SARI	Yes	Yes
**Sampling algorithm for RSV**				
**Information available**	Unique patient identifier;Age or date of birth;Sex;Geographical information;Date of clinical onset;Date of sampling;Date of diagnosis;Date of notification to surveillance organisation;Clinical symptoms;Immunosuppressive medication or condition;Chronic lung disease;Other pathogens detected	Unique patient identifier;Age or date of birth;Sex;Geographical information;Date of clinical onset;Date of sampling;Date of diagnosis;Date of notification to surveillance organisation; Clinical symptoms; Immunosuppressive medication or condition;Chronic lung disease;Other pathogens detected	Age or date of birth;Sex;Geographical information;Date of diagnosis	Age or date of birth;Sex;Geographical information;Date of diagnosis
**Denominator known**				No, only number of tests done by NRL, not all labs

Bulgaria

	Sentinel	Non-sentinel
**Type of health facilities**	GPs	Hospitals and GPs
**Set up**	Part of influenza surveillance	Part of influenza surveillance
**Period**	Week 40 to 20	All year round
**Frequency**	Weekly	Weekly
**Aggregation level**	aggregated	aggregated
**Population covered**	5,3%	
**Year introduced**	2001	1958
**Eligible for sampling**	ARI	ILI, SARI
**Sampling algorithm for RSV**	Yes	No
**Information available**	Yes	yes
**Denominator known**		no

Croatia

	Sentinel	Non-sentinel
**Type of health facilities**	None	Labs (All respiratory specimens sent to National Virus Reference Laboratory)
**Set up**		Through RSV-related studies
**Period**		All year
**Frequency**		weekly
**Aggregation level**		Case-based
**Population covered**		20%
**Year introduced**		1980
**Eligible for sampling**		No
**Sampling algorithm for RSV**		No
**Information available**		Age or date of birth;Sex;Geographical information;Hospitalization
**Denominator known**		No

Cyprus

	Sentinel	Non-sentinel
**Type of health facilities**	None	Hospitals (PICU/paediatric departments) unofficial system that captures most severe cases
**Set up**		Part of influenza surveillance
**Period**		Week 40-20
**Frequency**		Weekly
**Aggregation level**		Case-based
**Population covered**		Population<15 yrs
**Year introduced**		2015
**Eligible for sampling**		No. Left to clinical definition of paediatrician
**Sampling algorithm for RSV**		No
**Information available**		Unique patient identifier;Age or date of birth;Sex;Geographical information;Date of sampling;Clinical symptoms;Hospitalization
**Denominator known**		Yes

Czech Republic

	Sentinel	Non-sentinel
**Type of health facilities**	GP	Lab
**Set up**	Part of influenza surveillance	Part of influenza surveillance
**Period**	Week 40-20	Week 40-20 or all year around
**Frequency**	Weekly	Weekly
**Aggregation level**	Aggregated	Aggregated
**Population covered**		
**Year introduced**	From season 2013/2014 - introduction of PCR for sentinel samples, until then virus isolation and serology were used	
**Eligible for sampling**	ILI/ARI	ILI and ARI
**Sampling algorithm for RSV**	The same sampling strategy as for flu	The same sampling strategy as for flu
**Information available**	Unique patient identifier; Age or date of birth; Sex; Geographical information;Date of clinical onset; Date of sampling, Symptoms, Diagnosis	-
**Denominator known**		

Denmark

	Sentinel	Non-sentinel
**Type of health facilities**	GPs	Labs
**Set up**	Part of influenza surveillance	Notifications through national register
**Period**	Week 40-20	All year round
**Frequency**	Weekly	Weekly
**Aggregation level**	Aggregated	Aggregated
**Population covered**	1-5%	x
**Year introduced**	2009	2015
**Eligible for sampling**	ILI	No
**Sampling algorithm for RSV**	No	No
**Information available**	Unique patient identifier;Age or date of birth;Sex;Geographical information;Date of clinical onset;Date of sampling; Other: other data can be found in the national registries	Unique patient identifier;Age or date of birth;Sex;Geographical information;Date of clinical onset;Date of sampling;Date of diagnosis;Date of notification to surveillance organisation;Source of notification;Hospitalization;Premature birth;RSV related death;Other pathogens detected
**Denominator known**		Yes

Estonia

	Sentinel	Non-sentinel
**Type of health facilities**	GPs/Polyclinic/family doctor	Labs
**Set up**	Part of influenza surveillance	Other surveillance
**Period**	All year round	All year round
**Frequency**	Weekly	Weekly
**Aggregation level**	Aggregated by age group	Aggregated
**Population covered**	14%	x
**Year introduced**	2006	1965
**Eligible for sampling**	ILI	No
**Sampling algorithm for RSV**	No	x
**Information available**		
**Denominator known**		Yes

Finland

	Sentinel	Non-sentinel	
**Type of health facilities**	GPs	Hospitals (ICUs)	Labs
**Set up**	Part of influenza surveillance	Part of influenza surveillance	Part of other surveillance
**Period**	All year round	All year round	All year round
**Frequency**	Weekly	Weekly	
**Aggregation level**	Aggregated	Aggregated	
**Population covered**			
**Year introduced**	2005	2013	1995
**Eligible for sampling**	No	No	No
**Sampling algorithm for RSV**	No		
**Information available**	Unique patient identifier; Age or date of birth; Sex; Geographical information; Date of clinical onset; Date of sampling; Immunosuppressive medication or condition; Chronic lung disease; Hospitalization; Other pathogens detected	Unique patient identifier; Age or date of birth; Sex; Geographical information; Date of clinical onset; Date of sampling; Immunosuppressive medication or condition; Chronic lung disease; Hospitalization; Other pathogens detected	Unique patient identifier; Age or date of birth; Sex; Geographical information; Date of sampling; Date of notification to surveillance organisation; Source of notification
**Denominator known**		No	No

France

	Sentinel		Non-sentinel
**Type of health facilities**	Hospital labs (Renal)	GPs	Emergency medical visits (home (SOS Médecins) and in hospital (OSCOUR©)
**Set up**	Part of other surveillance	Part of other surveillance	Syndromic surveillance
**Period**	Week 40 to 20	Week 40 to 20	Week 40 to 20
**Frequency**	Weekly	Weekly	Weekly
**Aggregation level**	Aggregated	Aggregated	Aggregated
**Population covered**	All ages	All ages	Infants ≤ 2 years
**Year introduced**			2009
**Eligible for sampling**	ARI	ILI	Bronchiolitis in emergency visits and hospitalization for clinical bronchiolitis
**Sampling algorithm for RSV**		The same sampling strategy as for flu	x
**Information available**	Number of samples positive for RSV		Age;Sex;Geographical information : region;Hospitalization
**Denominator known**			No

Germany

	Sentinel	Non-sentinel
**Type of health facilities**	GPs	Other
**Set up**	Part of influenza surveillance	Through RSV-related studies
**Period**	All year	
**Frequency**	Weekly	
**Aggregation level**	Case-based	
**Population covered**	0-1%	
**Year introduced**	2010	
**Eligible for sampling**	ILI/ARI	
**Sampling algorithm for RSV**	No	
**Information available**	Age or date of birth;Sex;Geographical information; Date of clinical onset; Date of sampling; Date of diagnosis; Clinical symptoms; Immunosuppressive medication or condition; Chronic lung disease	
**Denominator known**		No

Greece

	Sentinel	Non-sentinel
**Type of health facilities**	None	Labs
**Set up**		Part of other surveillance
**Period**		All year round
**Frequency**		
**Aggregation level**		
**Population covered**		
**Year introduced**		
**Eligible for sampling**		
**Sampling algorithm for RSV**		
**Information available**		Unique patient identifier; Age or date of birth; Sex; Date of sampling;Date of diagnosis; Hospitalization time; RSV genotype
**Denominator known**		Yes: The total number of samples tested for RSV

Hungary

	Sentinel	Non-sentinel
**Type of health facilities**	GPs, Paediatric practice	Hospitals
**Set up**	Part of influenza surveillance	Part of influenza surveillance between 40-20 weeks
**Period**	week 40 to 20	All year round
**Frequency**	weekly	Weekly
**Aggregation level**	Case-based	Case-based
**Population covered**	>20%	
**Year introduced**	2005	2005
**Eligible for sampling**	Yes	Yes
**Sampling algorithm for RSV**	ILI	SARI
**Information available**	Yes	Yes
**Denominator known**		No

Iceland

	Sentinel	Non-sentinel	
**Type of health facilities**	None	GPs/hospitals	Labs
**Set up**		Part of other surveillance	Part of other surveillance
**Period**		All year round	All year round
**Frequency**		weekly	weekly
**Aggregation level**		case-based	case-based
**Population covered**			
**Year introduced**		2011	2011
**Eligible for sampling**		ICD-10	Testing based on clinical judgement
**Sampling algorithm for RSV**		No	No
**Information available**		Unique patient identifier;Age or date of birth;Sex;Geographical information;Date of sampling;Date of notification to surveillance organisation;Source of notification	Age or date of birth;Sex;Geographical information;Date of sampling;Date of notification to surveillance organisation;Source of notification
**Denominator known**		No	Yes

Ireland

	Sentinel	Non-sentinel	
**Type of health facilities**	GPs	Labs (All respiratory specimens sent to National Virus Reference Laboratory)	Notification system (all RSV detections, including outbreaks)
**Set up**	Part of influenza surveillance	Part of influenza surveillance	Specifically for RSV
**Period**	All year	All year	All year
**Frequency**	Weekly	Weekly	Weekly
**Aggregation level**	Case-based	Case-based	Case-based
**Population covered**	6%	x	x
**Year introduced**	2000	2000	2012
**Eligible for sampling**	ILI	No	Yes
**Sampling algorithm for RSV**	No	No	No
**Information available**	Unique patient identifier;Age or date of birth;Sex;Geographical information;Date of clinical onset;Date of sampling;Date of notification to surveillance organisation;Source of notification;Other pathogens detected;Other: influenza vaccination status, date of influenza vaccination, exposure to antivirals, is the patient in a risk group for flu. All sentinel specimens are tested for influenza, RSV, parainfluenza viruses 1-4, human metapneumovirus and adeno viruses	Unique patient identifier;Age or date of birth;Sex;Geographical information;Date of sampling;Date of notification to surveillance organisation;Source of notification;Other pathogens detected	Unique patient identifier;Age or date of birth;Sex;Geographical information;Date of clinical onset;Date of sampling;Date of diagnosis;Date of notification to surveillance organisation;Source of notification
**Denominator known**		Yes	Irish population

Italy

	Sentinel	Non-sentinel
**Type of health facilities**	None	None
**Set up**		
**Period**		
**Frequency**		
**Aggregation level**		
**Population covered**		
**Year introduced**		
**Eligible for sampling**		
**Sampling algorithm for RSV**		
**Information available**		
**Denominator known**		

Latvia

	Sentinel	Non-sentinel
**Type of health facilities**	GPs/family doctor	Hospitals
**Set up**	Part of influenza surveillance	Part of other surveillance
**Period**	Week 40-20	All year round
**Frequency**	Weekly	Weekly
**Aggregation level**	Case-based	Aggregated
**Population covered**	0-1%	x
**Year introduced**	2011	1993
**Eligible for sampling**	ILI	Testing based on clinical judgement
**Sampling algorithm for RSV**	No	No
**Information available**	Unique patient identifier;Age or date of birth;Sex;;Date of clinical onset; Clinical symptoms; Diagnosis; Date of sampling; Source of notification; Other pathogens detected; Influenza vaccination status; Date of influenza vaccination; Exposure to antivirals	Unique patient identifier;Age or date of birth;Sex;Date of clinical onset;Date of sampling
**Denominator known**		No

Lithuania

	Sentinel	Non-sentinel
**Type of health facilities**	None	None
**Set up**		
**Period**		
**Frequency**		
**Aggregation level**		
**Population covered**		
**Year introduced**		
**Eligible for sampling**		
**Sampling algorithm for RSV**		
**Information available**		
**Denominator known**		

Luxembourg

	Sentinel	Non-sentinel
**Type of health facilities**	None	None
**Set up**		
**Period**		
**Frequency**		
**Aggregation level**		
**Population covered**		
**Year introduced**		
**Eligible for sampling**		
**Sampling algorithm for RSV**		
**Information available**		
**Denominator known**		

Malta

	Sentinel	Non-sentinel
**Type of health facilities**	None	Hospital labs
**Set up**		Part of influenza surveillance
**Period**		All year round
**Frequency**		Weekly
**Aggregation level**		Case-based
**Population covered**		
**Year introduced**		2013
**Eligible for sampling**		ILI
**Sampling algorithm for RSV**		
**Information available**		Unique patient identifier;Sex;Date of sampling;Date of diagnosis
**Denominator known**		

The Netherlands

	Sentinel	Non-sentinel	
**Type of health facilities**	GPs	Labs	Hospitals
**Set up**	Part of influenza surveillance	Notifications	Network of PICUs including lab report*
**Period**	All year round	All year round	Week 40 to 20
**Frequency**	Weekly	Weekly	Weekly
**Aggregation level**	Case-based	Aggregated	Aggregated
**Population covered**	0-1%	~80%	x
**Year introduced**	1992	1964	2016
**Eligible for sampling**	ILI and ARI	No	No
**Sampling algorithm for RSV**	No	x	x
**Information available**	Unique patient identifier;Age or date of birth;Sex;Geographical information;Date of clinical onset;Date of sampling;Date of diagnosis;Date of notification to surveillance organisation;Source of notification;Clinical symptoms;Immunosuppressive medication or condition;Chronic lung disease;Other pathogens detected	Date of diagnosis;Date of notification to surveillance organisation;Source of notification (which lab)	Hospitalization;Other pathogens detected;Other: week of admission to PICU, four age categories for SARI patients
**Denominator known**		No	No

- Note: this surveillance has only be operational during season 2016-2017

Norway

	Sentinel	Non-sentinel
**Type of health facilities**	None	Labs
**Set up**		part of other surveillance
**Period**		All year round
**Frequency**		Monthly
**Aggregation level**		Aggregated
**Population covered**		
**Year introduced**		1969
**Eligible for sampling**		No
**Sampling algorithm for RSV**		No
**Information available**		
**Denominator known**		No

Poland

	Sentinel	Non-sentinel	
**Type of health facilities**	GPs	Hospitals	Labs
**Set up**	Part of influenza sentinel surveillance	Part of influenza surveillance	Part of influenza surveillance
**Period**	All year round	All year round	All year round
**Frequency**	Weekly	Weekly	Weekly
**Aggregation level**	Case-based	Case-based	Case-based
**Population covered**	1-5%	x	x
**Year introduced**	2004	2004	2004
**Eligible for sampling**	ILI	Do not know	Do not know
**Sampling algorithm for RSV**	Do not know	x	x
**Information available**	Age or date of birth;Geographical information;Date of notification to surveillance organisation	Age or date of birth;Geographical information;Date of notification to surveillance organisation	Geographical information;Date of notification to surveillance organisation
**Denominator known**		Do not know	Do not know

Portugal

	Sentinel	Non-sentinel
**Type of health facilities**	GPs and emergency units	Hospital labs
**Set up**	Part of influenza surveillance	Part of influenza surveillance
**Period**		Week 40-20
**Frequency**		Weekly
**Aggregation level**		Case-based
**Population covered**		x
**Year introduced**		2010
**Eligible for sampling**	Influenza negative cases	ILI/ARI
**Sampling algorithm for RSV**		No
**Information available**		Age or date of birth;Sex; Geographical information;Date of sampling; Date of notification to surveillance organisation; Source of notification; Hospitalization; Other pathogens detected; Death
**Denominator known**		Yes: The actual denominator is the total number of samples tested for influenza. It will be possible to have a more accurate number of samples tested only for RSV, only for influenza and tested for both viruses.

Romania

	Sentinel*	Non-sentinel
**Type of health facilities**		Hospitals (ICU/paediatric department and infectious disease ward)
**Set up**		Part of influenza surveillance
**Period**		Week 40-20
**Frequency**		Weekly
**Aggregation level**		Case-based
**Population covered**		x
**Year introduced**		2010
**Eligible for sampling**		SARI, negative for influenza (sporadically)
**Sampling algorithm for RSV**		No
**Information available**		Unique patient identifier;Age or date of birth;Sex;Geographical information;Date of clinical onset;Date of sampling;Date of diagnosis;Source of notification;Hospitalization
**Denominator known**		No

*sentinel surveillance between 2010/2011 and 2013/2014

Spain

	Sentinel	Non-sentinel
**Type of health facilities**	None	Labs
**Set up**		RSV detections. It was set up as a complementary system of Influenza surveillance
**Period**		Week 40-20
**Frequency**		Weekly
**Aggregation level**		Aggregated
**Population covered**		Not known
**Year introduced**		2006
**Eligible for sampling**		No
**Sampling algorithm for RSV**		No
**Information available**		Weekly RSV detections
**Denominator known**		Total number of samples tested for viral diagnostic purposes

Slovakia

-

	Sentinel		Non-sentinel
**Type of health facilities**	GPs/paediatric practice	Hospital (ICU)	None
**Set up**	Part of influenza surveillance	Part of influenza surveillance	
**Period**	All year round	All year round	
**Frequency**	Weekly	Weekly	
**Aggregation level**	Case-based	Case-based	
**Population covered**	not known	100%	
**Year introduced**	2002	2006	
**Eligible for sampling**	No	No	
**Sampling algorithm for RSV**	No	No	
**Information available**	Yes	Yes	
**Denominator known**			

Slovenia

	Sentinel	Non-sentinel
**Type of health facilities**	Paediatricians, School doctors and General Practitioners	Labs
**Set up**	Part of influenza surveillance	Specifically for RSV
**Period**	All year round	All year round
**Frequency**	Weekly	Weekly
**Aggregation level**	Case-based	Aggregated
**Population covered**	4%	100%
**Year introduced**	1999	2006
**Eligible for sampling**	ILI	ARI
**Sampling algorithm for RSV**	No	No
**Information available**	Patient specific data: age, gender, place of residence, onset, date of specimen taken, influenza vaccination status, clinical symptoms	Geographical information;Source of notification; Aggregated data are reported on weekly basis with no exact date of sampling or clinical onset.
**Denominator known**		Yes

Sweden

	Sentinel	Non-sentinel	
**Type of health facilities**	None	Labs and paediatric clinics	Hospital (ICU excluding NICU)
**Set up**		Part of influenza surveillance	Other surveillance
**Period**		Week 40 to 20	All year round
**Frequency**		Weekly	Weekly
**Aggregation level**		Case-based	Aggregated
**Population covered**		Total population	Total population
**Year introduced**		1999	2008
**Eligible for sampling**		No	No
**Sampling algorithm for RSV**		x	Unknown
**Information available**		Age or date of birth;Sex;Geographical information;Date of diagnosis;Date of notification to surveillance organisation;Source of notification;Other: Info from of ICU of RSV cases	Aggregated reports by age group, gender, reporting ICU, geographic region, primary vs other diagnosis of RS pneumonia, type of treatment, and other data.
**Denominator known**		Yes	Yes

United Kingdom

	Sentinel	Non-sentinel
**Type of health facilities**	GPs	Lab and Hospitals (all hospital testing)
**Set up**	Part of influenza surveillance	Part of other surveillance
**Period**	Week 40 to week 20	All year round
**Frequency**	Weekly	Weekly
**Aggregation level**	Aggregated	Aggregated
**Population covered**	0-1%	
**Year introduced**	2000	2007
**Eligible for sampling**	No	No
**Sampling algorithm for RSV**	A random sample of including young children (<5 years) presenting with acute bronchitis/bronchiolitis (up to a maximum of 5 per week).	
**Information available**	Unique patient identifier;Age or date of birth;Sex;Geographical information;Date of clinical onset;Date of sampling;Date of diagnosis;Date of notification to surveillance organisation;Source of notification;Clinical symptoms;Other pathogens detected	Age or date of birth;Sex;Geographical information;Date of sampling;Date of diagnosis;Date of notification to surveillance organisation;Source of notification;Clinical symptoms
**Denominator known**		No
